# Serum IgG Responses to gp15 and gp40 Protein-Derived Synthetic Peptides From *Cryptosporidium parvum*

**DOI:** 10.3389/fcimb.2021.810887

**Published:** 2022-01-19

**Authors:** Alejandro Urrea-Quezada, Ruben Balmaceda-Baca, Adriana Garibay, Jesús Hernández, Olivia Valenzuela

**Affiliations:** ^1^ Departamento de Ciencias Químico Biológicas, Universidad de Sonora, Hermosillo, Mexico; ^2^ Laboratorio de Inmunología, Centro de Investigación en Alimentación y Desarrollo A. C., Hermosillo, Mexico

**Keywords:** human cryptosporidiosis, emerging parasites, humoral response, synthetic peptides, IgG

## Abstract

*Cryptosporidium* spp. are responsible for moderate to severe diarrhea, mainly in children and immunocompromised patients. Using ELISA, the recognition of synthetic peptides generated from the sequences of the *Cryptosporidium parvum* gp40 and gp15 proteins by serum IgM and IgG antibodies from patients infected (cases) with *Cryptosporidium hominis*, *C. parvum*, and *Cryptosporidium canis*, and uninfected individuals (controls) was evaluated. A statistically significant difference (p = 0.0025) was found in terms of the recognition of peptides A133 and A32 between cases and controls. Additional studies are necessary to understand the potential of these peptides as vaccine candidates.

## Introduction

*Cryptosporidium* spp. of the phylum Apicomplexa are protozoan parasites of medical and veterinary importance worldwide because they are causal agents of moderate to severe diarrhea, with higher prevalence in developing countries (Chalmers and Giles, 2010; Bouzid et al., 2013). Children under 5 years old, and immunocompromised people, are the most susceptible to cryptosporidiosis (Sarkar et al., 2012). In immunocompetent people, the disease can occur asymptomatically; if there are symptoms, they are self-limited with a duration of 1–2 weeks (Widerström et al., 2014). The Food and Drug Administration (FDA) has approved nitazoxanide as a drug against cryptosporidiosis with an effectiveness of 56%–96% in immunocompetent people (Checkley et al., 2015).

Recent efforts to develop a vaccine against *Cryptosporidium* spp. have focused on the identification and characterization of immunogenic proteins (GP900, 27-kD, gp15/CP15, gp40, CSL) involved in the adhesion and invasion of *Cryptosporidium* merozoites and sporozoites (Frost et al., 2005; Ajjampur et al., 2011; Allison et al., 2011; Avendaño et al., 2018). One of the most studied proteins is GP60 or gp40/15, which is located in the apical complex of the parasite (Cevallos et al., 2000; Borad and Ward, 2010). Anti-gp15 and anti-gp40 antibodies are associated with protection in humans (Ajjampur et al., 2011; Allison et al., 2011). Synthetic peptide-based vaccines versus attenuated pathogen vaccines or DNA vaccines are less difficult to manufacture and require less stringent storage conditions (Avendaño et al., 2018); therefore, synthetic peptides with potential as vaccine candidates are of great interest. The objective of this study was to evaluate humoral immunity, as demonstrated in the recognition of synthetic peptides of the *Cryptosporidium parvum* gp15 and gp40 proteins by serum IgM and IgG antibodies from patients infected (cases) with *Cryptosporidium hominis*, *C. parvum*, and *Cryptosporidium canis*, and uninfected individuals (controls).

## Materials and Methods

### Statements of Ethics

The protocol was registered in the Secretaría de Salud del Estado de Sonora (project No. 196) and approved by the Ethics Committee of the Hospital Infantil del Estado de Sonora (HIES) and the Centro Médico Dr. Ignacio Chávez (CMDICH) (CEI-09-2015). Informed consent was obtained from each patient after the objectives of the study were clearly explained; in the case of minors, informed consent was also obtained from the parent or guardian. The clinical characteristics of the patients were obtained with prior authorization from the patients’ clinical files at each hospital.

### Subjects (Control and Those With Cryptosporidiosis)

The inclusion criteria of the patients (case) for the study were being positive for *Cryptosporidium* by Kinyoun staining, male or female, of any age and with or without clinical symptoms of acute gastroenteritis (AGE). The group of cases consisted of 39 children from 5 months to 9 years of age (51.3% male). Patients in this group were carriers of *Cryptosporidium* spp., as diagnosed by microscopic observation of oocysts (Henriksen and Pohlenz, 1981) and molecular characterization *via* nested analysis (PCR sequencing) of small subunit rRNA (SSU rRNA) and GP60 (Valenzuela et al., 2014; Gonzalez-Diaz et al., 2016; Urrea-Quezada et al., 2018); 89.7% of the cryptosporidiosis cases (35/39) were recruited from the HIES (**Supplementary Table 1**). The most frequent admission diagnoses (87.2%) were acute gastroenteritis (AGE), malnutrition (25.6%), and dehydration (15.4%); however, three patients had an admission diagnosis that was unrelated to a gastrointestinal disorder: acute otitis, pulmonary tuberculosis, pneumonia, and bronchiolitis. Ten percent of the cases (4/39) were recruited from the CMDICH. The admission diagnosis of each of them was different; one case went to the doctor for a general check-up, two for AGE, and the last for a urinary tract infection (UTI) (**Table 1**).

**Table 1:** *Cryptosporidium* species and subtypes identified from Sonora, Mexico.

Species/Genotype (n)	Subtype (n)	Age	Gender	Clinical symptoms
*C. hominis* (13)	IaA14R3 (2)	5M	Female	SM, FT, F, P, C
		1Y	Male	SM, D, DH, F
	IaA14R11 (1)	1Y	Female	LM, D, DH, V, FT
	IaA15R3 (3)	1Y	Female	LM, D, FT, AP, V, F, E
		1Y	Male	D, DH
		1Y	Male	B
	IbA12G3 (3)	9M	Female	D, AP, V, F
		2Y	Female	NS
		4Y	Female	AP, AOM, HD
	IeA11G3T3 (4)	7M	Female	LM, D, DH, P
		1Y		PTB, AP
		3Y	Male	SM, D, V, HPT, H
		3Y	Female	D
			Male	
*C. parvum* (19)	IIaA15G2R1 (18)	1Y	Male	D
		1Y	Female	D, AP, F, HD
		1Y	Female	D, FT, V, F
		2Y	Female	D, FT, AP, V, F
		2Y	Male	D, V, F, AOM
		2Y	Male	AP, FT
		2Y	Male	D, F, IDA
		3Y	Female	D, AP
		3Y	Female	D, FT, AP, V, HD
		3Y	Male	D, FT, AP, V
		3Y	Male	D, AP, DH, V
		4Y	Female	AP, FT
		4Y	Male	D
		4Y	Female	D, V
		6Y	Male	D, HD, F, V, FT
		6Y	Male	SM, D, HIV, P, PTB, CO
		8Y	Female	FT, AP, V, F, UTI, HD
		9Y	Male	HIV, SM, D, CO
	IIcA5G3a (1)	1Y	Female	SM, D, V, F
*C. canis* (1)	–	2Y	Male	D
*C. canis/C. hominis* (1)	–	1Y	Male	D, AP, F, V, HD, FT
*C. parvum/C. hominis* (3)	IbA12G3	5M	Female	LM, D, DH
	IIaA15G2R1	7Y	Male	D, FT, AP, V
	IIcA5G3a	7Y	Female	NS
*Cryptosporidium* spp. (2)	–	1Y	Male	AS
	–	3Y	Male	D, AP, F, FT

Y, years; M, months; SM, severe malnutrition; MM, moderate malnutrition; LM, mild malnutrition; D, diarrhea; DH, dehydration; V, vomiting.; F, fever; AP, abdominal pain; IDA, iron deficiency anemia; H, hypothyroidism; P, pneumonia; CO, candidiasis oral; AOM, acute otitis media; S, sinusitis; AR, allergic rhinitis; PTB, pulmonary tuberculosis; C, conjunctivitis; UTI, urinary tract infection; E, epilepsy; HIV, HIV positive; HPT, hepatitis; HD, headache; FT, flatulence; B, Bronchitis; NS, no symptoms; NA, not available.

The inclusion criterion for the controls was being negative for *Cryptosporidium* as determined microscopically and *via* molecular techniques. This group includes male or female individuals of any age with or without clinical symptoms of acute gastroenteritis (AGE) and others symptoms not related to AGE. The control group comprised 90 subjects from 1 month to 65 years old who were *Cryptosporidium* negative, 47.8% of whom were female (**Supplementary Table 1**). All of the included participants reside in urban areas of the state of Sonora. Thirty-four of the subjects were recruited from the HIES; all of the subjects were under 15 years of age, and 88% of them (30/34) had admission diagnoses of various infections: respiratory tract (16/30) and GEA (6/30) (**Supplementary Table 1**). Thirty-one of the controls were recruited from the CMDICH (1–65 years), and the admission diagnosis was not determined in any of them. In addition, a group of 21-year-old volunteers were included in the study, 15 of whom were apparently healthy and 10 of whom presented with clinical manifestations (diarrhea and abdominal pain).

### Serum and Stool Samples

Serum and fecal samples of each participant were sent to the Laboratory of Parasitology of the Departamento de Ciencias Químico Biológicas of the Universidad de Sonora. Feces was used for the microscopic diagnosis of intestinal parasites and molecular characterization of *Cryptosporidium*; serum was used for the determination of whether there was humoral recognition (IgM and IgG) of the peptides using ELISA. The fecal samples were stored at 4°C, and the serum samples were stored at −20°C until further analysis.

Sera from four apparently healthy patients (two toddlers and two adults) without cryptosporidiosis who did not show recognition for the recombinant proteins (rCpGP15, rCpGP40, and rChGP40) and peptides (A109, A133, V30, A33, and R61) were used as negative controls. For the positive controls, we used two groups: a group composed of three children infected with *C. parvum* and a group composed of three children infected with *C. hominis*; all the positive controls had strong recognition for all the peptides and recombinant proteins (data not shown). All the patient samples were tested in duplicate. A sample was considered positive when the absorbance was above the cutoff, which consisted of the mean plus two standard deviations of the negative control.

### Detection of *Cryptosporidium* Oocysts

Human fecal samples were collected from children and adults from Hermosillo, Sonora, México. Oocysts were detected by the modified Ziehl–Neelsen (MZN) method. The oocysts were stained pink on a pale green background, appeared round or spherical in shape, and were 4–6 µm in diameter (Henriksen and Pohlenz, 1981).

### DNA Extraction

DNA extraction from the stool samples was performed with the ZR Fecal DNA MiniPrep Extraction Kit (Zymo Research Corp., Irvine, CA) according to the manufacturer’s instructions after five freeze (−20°C) and thaw (95°C) cycles, and the extracted DNA was stored at −20°C until further processing (Valenzuela et al., 2014).

### Molecular Diagnosis of Cryptosporidiosis

Molecular characterization was performed by analyzing the sequences of the nested PCR products for the SSU rRNA (826–864 bp) and GP60 (880–900 bp) genes (Valenzuela et al., 2014). To identify patients with possible coinfections of different *Cryptosporidium* species, real-time PCR (qPCR) analysis was performed using the Chos-1 and Cops-2 genes of *C. hominis* and *C. parvum*, respectively (Bouzid et al., 2016).

### Peptide Design

The peptides were designed from the sequences of the *C. parvum* gp15 protein and gp40 protein using the Chromas Lite version 2.1 program. The Clustalw program was used to align multiple sequences and detect differences and similarities among the different DNA sequences of the samples obtained. Finally, to compare the sequences identified in this work with others reported worldwide, the Blastn program was used. The sequences were synthesized by GenScript (Piscataway, NJ, USA) to crude purity and adjusted to a concentration of 1 mg/ml in water.

### Enzyme-Linked Immunosorbent Assays for the Anti-gp15 IgG and Anti-gp40 IgG Protein-Derived Synthetic Peptides From *Cryptosporidium parvum*

The serum samples were used to detect peptide-specific antibodies using enzyme-linked immunosorbent assay (ELISA) modified from Priest (Priest et al., 2005). Briefly, 96-well clear flat bottom microplates (Corning, Inc., New York, NY, USA) were coated with 0.5 µg of each peptide in carbonate buffer 0.1 M, pH 9.6. The 96-well plates were coated overnight at 4°C. Excess antigen was removed with three washes with 0.05% Tween 20–PBS [pH 7.4; phosphate-buffered saline (PBS)]; nonspecific binding was blocked with a 5% nonfat dry milk solution for 4 h at room temperature. After three washes with Tween 20–PBS solution, the wells were incubated with serum diluted 1:100 in PBS for 1 h at room temperature. After washing, goat anti-human IgG and IgM with peroxidase (dilutions of 1:1,000 and 1:500, respectively) (Southern Biotech Associates, Inc., Birmingham, AL, USA) were added and incubated for 1 h at room temperature. After being washed, the wells were incubated with the TMB substrate solution (Sigma, St. Louis, MO, USA) at room temperature in the dark, and the reaction was stopped after 5 min with H_2_S0_4_. Absorbance was read on a microplate absorbance reader at 450 nm (iMark Bio-Rad).

### Statistical Analysis

Data were analyzed using GraphPad Prism 5 for Windows (GraphPad Software, Inc., San Diego, CA). The Mann–Whitney U-test was used to determine differences between the patient group and the control group.

## Results

### Species of *Cryptosporidium* Identified

Of the 39 cases with cryptosporidiosis included in this study, *C. parvum* (19/39) was identified in 48.7%, *C. hominis* (13/39) in 33.3%, and *C. canis* in 2.6% (1/39) by analyzing the sequence of the SSU rRNA gene. In 7.7% of the cases (3/39), a coinfection of *C. parvum* and *C. hominis* was determined by qPCR, and in 2.6% of the cases (1/39), a coinfection of *C. canis* and *C. hominis* was determined (**Table 1**).

### Peptide Antigens of the *Cryptosporidium parvum* gp15 and gp40 Proteins

Five peptides from *C. parvum*, four peptides of the gp15 protein (A109, A133, A32, and R61), and one peptide of the gp40 protein (V30) were designed and used in this study (**Supplementary Table 2**).

### Seroprevalence to Peptides of the *Cryptosporidium parvum* gp15 and gp40 Proteins in Cases and Controls

**Figure 1:**
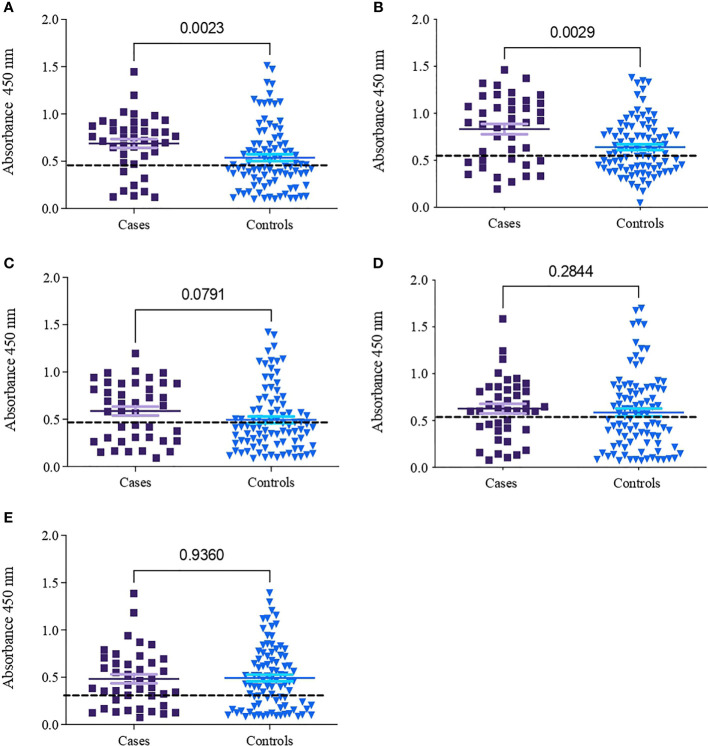
Absorbances obtained between cases and controls for four peptides of the *Cryptosporidium parvum* proteins gp15 and gp40. **(A)** A133; cutoff line (0.46). **(B)** A32; cutoff line (0.57). **(C)** R61; cutoff line (0.47). **(D)** A109; cutoff line (0.55). **(E)** V30; cutoff line (0.33).

**Table 2** shows the seroprevalence result of the IgG response of infected patients (cases) and noninfected individuals (controls) to peptides generated from gp15 sequences (A109, A133, A32, and R61) and a gp40 sequence (V30) from *C. parvum*. The seroprevalences of the cases for peptides A133 and A32 were 84.6% and 74.4%, respectively. The peptides A133 and A32 showed a statistically significant difference (p <0.003) in the recognition of sera between cases and controls, with stronger recognition by the cases (**Figures 1A, B**). The recognition of peptides R61, A109, and V30 did not show a statistically significant difference (p >0.05) between cases and controls (**Figures 1C–E**).

**Table 2:** Seroprevalence of cryptosporidiosis in cases and controls.

Population	A109 % Prevalence	A133 % Prevalence	V30 % Prevalence	A32 % Prevalence	R61 % Prevalence
Controls (n = 90)	48.9 (44/90)	50.0 (45/90)	62.2 (56/90)	52.2 (47/90)	41.1 (37/90)
1 month–15 years (n = 43)	18.9 (17/90)	22.2 (20/90)	31.1 (28/90)	28.9 (26/90)	21.1 (19/90)
19–65 years (n = 35)	27.8 (25/90)	24.4 (22/90)	22.2 (20/90)	20.0 (18/90)	17.8 (16/90)
Cases (n = 39)	66.7 (26/39)	84.6 (33/39)	69.2 (27/39)	74.4 (29/39)	61.5 (24/39)
*C. parvum* (n = 19)	30.8 (12/39)	41.0 (16/39)	38.5 (15/39)	41.0 (16/39)	35.9 (14/39)
*C. hominis* (n = 13)	25.6 (10/39)	30.8 (12/39)	20.5 (8/39)	17.9 (7/39)	15.4 (6/39)
*C. canis* (n = 1)	0.0 (0/39)	2.6 (1/39)	0.0 (0/39)	0.0 (0/39)	2.6 (1/39)
*C. parvum/C. hominis* (n = 3)	2.6 (1/39)	2.6 (1/39)	2.6 (1/39)	7.7 (3/39)	2.6 (1/39)
*C. canis/C. hominis* (n = 1)	2.6 (1/39)	2.6 (1/39)	2.6 (1/39)	2.6 (1/39)	2.6 (1/39)
*Cryptosporidium* spp. (n=2)	5.1 (2/39)	5.1 (2/39)	5.1 (2/39)	5.1 (2/39)	5.1 (2/39)

In the control group, two age groups were observed: those who were younger than 15 years old and those who were older than 19 years old (**Table 2**). Statistically significant differences were not found for the recognition of peptides A133 and A32 by age group (p = 0.862, p = 0.082).

### Recognition of Peptides Among Infecting *Cryptosporidium* Species

The serum recognition (IgG) of the five peptides tested in this study by infecting genotype/species (*C. parvum*, *C. hominis*, and *C. canis*) is shown in **Supplementary Figure 1**, and no statistically significant difference was found (p > 0.4).

### IgM and IgG Response to A133 Peptide

**Supplementary Figures 2A, B** show a statistically significant difference between the IgM and IgG antibody titers both in cases (p < 0.0001) and controls (p < 0.0001) for the recognition of the A133 peptide. Similarly, after determining the cutoff (0.5), it was identified that 79.5% of the cases (31/39) (**Supplementary Figure 2C**) and 43.3% of the controls (39/90) were above the cutoff (**Supplementary Figure 2D**).

### IgM and IgG Response to A32 Peptide

**Supplementary Figures 3A, B** show that there was a statistically significant difference between the IgM and IgG antibody titers in both the cases (p = 0.0001) and controls (p = 0.0001) for the recognition of the A32 peptide; after determining the cohort line (0.55), it was identified that 74.4% of the cases (29/39) (**Supplementary Figure 3C**) and 54.4% of the controls (49/90) were above the cutoff (**Supplementary Figure 3D**).

The A32 and A133 peptides described here have been submitted to the Instituto Mexicano de la Propiedad Industrial to obtain their patents (MX/a/2019/013932 and MX/a/2019/013933).

## Discussion

Despite the design of the peptides tested, A109, A133, A32, and R61 were generated from the sequence of *C. parvum* gp15; after alignment with other reported sequences, peptides A109, A32, and R61 were found in *C. hominis* gp15. Therefore, these peptides are present in the gp15 region of both species (conserved epitopes), and no statistically significant differences were found according to the genotype/infecting species. However, for the A133 and V30 peptides, whose sequences were only aligned in isolates of *C. parvum*, there was no difference in recognition by cases according to the genotype/infecting species. In previous studies, the recognition of recombinant proteins of gp15 (rgp15) of *C. hominis* (Allison et al., 2011) and rgp40 of *C. parvum* (Ajjampur et al., 2011) by serum IgG has been observed. Additionally, statistically significant differences in the recognition of the antigens between cases and controls, and cross-reactivity between epitopes (peptides) of these antigens (rgp15Ch, rgp40Cp), have been observed (Ajjampur et al., 2011; Allison et al., 2011).

The recognition of these peptides determines whether the patient is or was infected by *Cryptosporidium*. Based on the way the peptides were designed, it was expected to find specific genotype/species recognition; however, in the cases included in the study, specific genotype/species recognition was not observed, perhaps because both the cases and controls had a previous encounter with *Cryptosporidium*. Additionally, the serum IgG titer towards three of the peptides (A109, V30, and R61) tested did not show differences between the cases and controls. In previous studies, it has been shown that multiple encounters with a parasite are required to generate a significant IgG titer (DuPont et al., 1995). Despite this, in this study, it was managed to generate two peptides, A133 and A32, from gp15 and gp40 of *C. parvum*, respectively; however, A133 and A32 were similar in terms of their amino acid sequences. In addition, it was determined that the recognition of A133 and A32 was significantly different between the cases and controls (p = 0.0023 and p = 0.0029, respectively); however, it was not possible to determine statistically significant differences (p > 0.05) in the recognition of the peptides by infecting species.

Using the peptides A133 and A32, an analysis of the serum IgM antibody was performed to determine whether cases with lower absorbance of IgG had higher absorbance of IgM based on the fact that those with an acute infection have higher IgM titers that decrease over the course of a few weeks, which is opposite of the trend of IgG. It was found that there were statistically significant differences (p < 0.0001) between the serum IgM and IgG antibodies in the cases and controls, which led it to suggest that, in most of these cases, the patients were not infected for the first time or had been infected for several weeks. It was observed that the controls that were above the cut-off had high levels of the IgG antibody; one explanation for this result is memory antibodies.

Our study reveals the ability of two peptides (A133 and A32) of the *C. parvum* gp15 protein to identify and discriminate between infected (cases) and uninfected (controls) individuals. These results agree with a previous report by Avendaño *et al.*; this study evaluated five synthetic peptides of the gp15 (Cp15) and CSL proteins. The results showed that all peptides can stimulate antibody production, but only two peptides neutralize parasites *in vitro* (Avendaño et al., 2018). In addition, another report showed that antibodies against anti-gp40/15 and anti-gp40 antibodies block *C. parvum* infection *in vitro* (Cui et al., 2020). These data suggest that regions on the gp15 protein of *C. parvum* induce a strong antibody response in infected individuals. These regions probably can induce antibodies able to neutralize the *Cryptosporidium*. These results can support these peptides in future research to develop a *Cryptosporidium* vaccine. Other studies evaluating the cellular immune response are required to understand better the immune response and the participation of these peptides.

## Data Availability Statement

The original contributions presented in the study are included in the article/**Supplementary Material**. Further inquiries can be directed to the corresponding author.

## Ethics Statement

The studies involving human participants were reviewed and approved by Ethics Committee of the Hospital Infantil del Estado de Sonora (HIES). Written informed consent to participate in this study was provided by the participants’ legal guardian/next of kin.

## Author Contributions

OV: conception, project administration, and supervision. AU-Q and VO: writing original draft. JH and OV: visualization. AU-Q, RB-B, AG, JH, and OV: investigation and data analysis. OV, JH, and AG: validation. AU-Q and RB-B: methodology. OV: resources. OV: funding acquisition. All authors contributed to the article and approved the submitted version.

## Funding

This work was supported by the Consejo Nacional de Ciencia y Tecnología (CONACyT), Fondo Sectorial de Investigación para la Educación (grant number 258454).

## Conflict of Interest

The authors declare that the research was conducted in the absence of any commercial or financial relationships that could be construed as a potential conflict of interest.

## Publisher’s Note

All claims expressed in this article are solely those of the authors and do not necessarily represent those of their affiliated organizations, or those of the publisher, the editors and the reviewers. Any product that may be evaluated in this article, or claim that may be made by its manufacturer, is not guaranteed or endorsed by the publisher.

## Supplementary Material

Supplementary Table 1 Distribution of cases and controls by hospital of origin.

Supplementary Table 2 Peptide antigens of the *Cryptosporidium parvum* gp15 and gp40 proteins.

Supplementary Figure 1 Recognition of peptides among infecting *Cryptosporidium* species. The line is cut-off (average plus two standard deviations) for each peptide obtained from the average of the absorbances of the controls. Cp, *C. parvum*; Ch, *C. hominis*; Cc, *C. canis*. **(A)** Peptide A109. **(B)** Peptide A133. **(C)** Peptide V30. **(D)** Peptide A32. **(E)** Peptide R61.

Supplementary Figure 2 IgM and IgG anti-*Cryptosporidium* A133 peptide. **(A)** Cases. **(B)** Controls. **(C)** Cases with IgG above the cutoff line (0.5). **(D)** Controls with IgG above the cut-off line (0.5). **(E)** Cases with IgG below the cutoff line (0.5). **(F)** Controls with IgG below the cutoff line.

Supplementary Figure 3 IgM and IgG anti-*Cryptosporidium* A32 peptide. **(A)** Cases. **(B)** Controls. **(C)** Cases with IgG above the cutoff line (0.556). **(D)** Controls with IgG above the cut-off line (0.05). **(E)** Cases with IgG below the cutoff line (0.05). **(F)** Controls with IgG below the cut-off line.
