# Supplementary material for: Serum IgG Responses to gp15 and gp40 Protein-Derived Synthetic Peptides From Cryptosporidium parvum
Source: Front Cell Infect Microbiol. 2022 Jan 19;11:810887. doi: 10.3389/fcimb.2021.810887 (PMC8807513; doi:10.3389/fcimb.2021.810887)
Supplement: Supplementary Table 1 — Distribution of cases and controls by hospital of origin. [file Table_1.docx]

|  | N | Sex | |
| --- | --- | --- | --- |
| Cases | 39 | F | M |
| CMDICH | 10.3% (4/39) | 25% (1/4) | 75% (3/4) |
| HIES | 89.7% (35/39) | 48.6 (17/35) | 51.4% (18/35) |
| Controls | 90 | F | M |
| CMDICH | 34.4% (31/90) | 48.4% (15/31) | 51.6% (16/31) |
| HIES | 37.8% (34/90) | 41.2% (14/34) | 58.8% (20/34) |
| Volunteers | 27.8% (25/90) | 56.0% (14/25) | 44.0% (11/25) |
