# Supplementary material for: Serum IgG Responses to gp15 and gp40 Protein-Derived Synthetic Peptides From Cryptosporidium parvum
Source: Front Cell Infect Microbiol. 2022 Jan 19;11:810887. doi: 10.3389/fcimb.2021.810887 (PMC8807513; doi:10.3389/fcimb.2021.810887)
Supplement: Supplementary Table 2 — Peptide antigens of the Cryptosporidium parvum gp15 and gp40 proteins. [file Table_2.docx]

| **Supplementary S2.** Peptide antigens of the *Cryptosporidium parvum* gp15 and gp40 proteins |
| --- |
| \| Peptide \| Start position \| Sequence \| End position \| \| --- \| --- \| --- \| --- \| \| V30 \| 30 \| VYAPIKDQTDPAPRYISG \| 47 \| \| A109 \| 109 \| ATVDLFAFTLDGGRRIE \| 125 \| \| A133 \| 133 \| ADKRSEYSLVADDKPFYT \| 151 \| \| A32 \| 32 \| ASKRDKYSLVADDKPFYT \| 49 \| \| R61 \| 61 \| RLNENGDLVDKDNTVLLK \| 78 \| |
