# Supplementary figures and images for: Serum IgG Responses to gp15 and gp40 Protein-Derived Synthetic Peptides From Cryptosporidium parvum
Source: Front Cell Infect Microbiol. 2022 Jan 19;11:810887. doi: 10.3389/fcimb.2021.810887 (PMC8807513; doi:10.3389/fcimb.2021.810887)

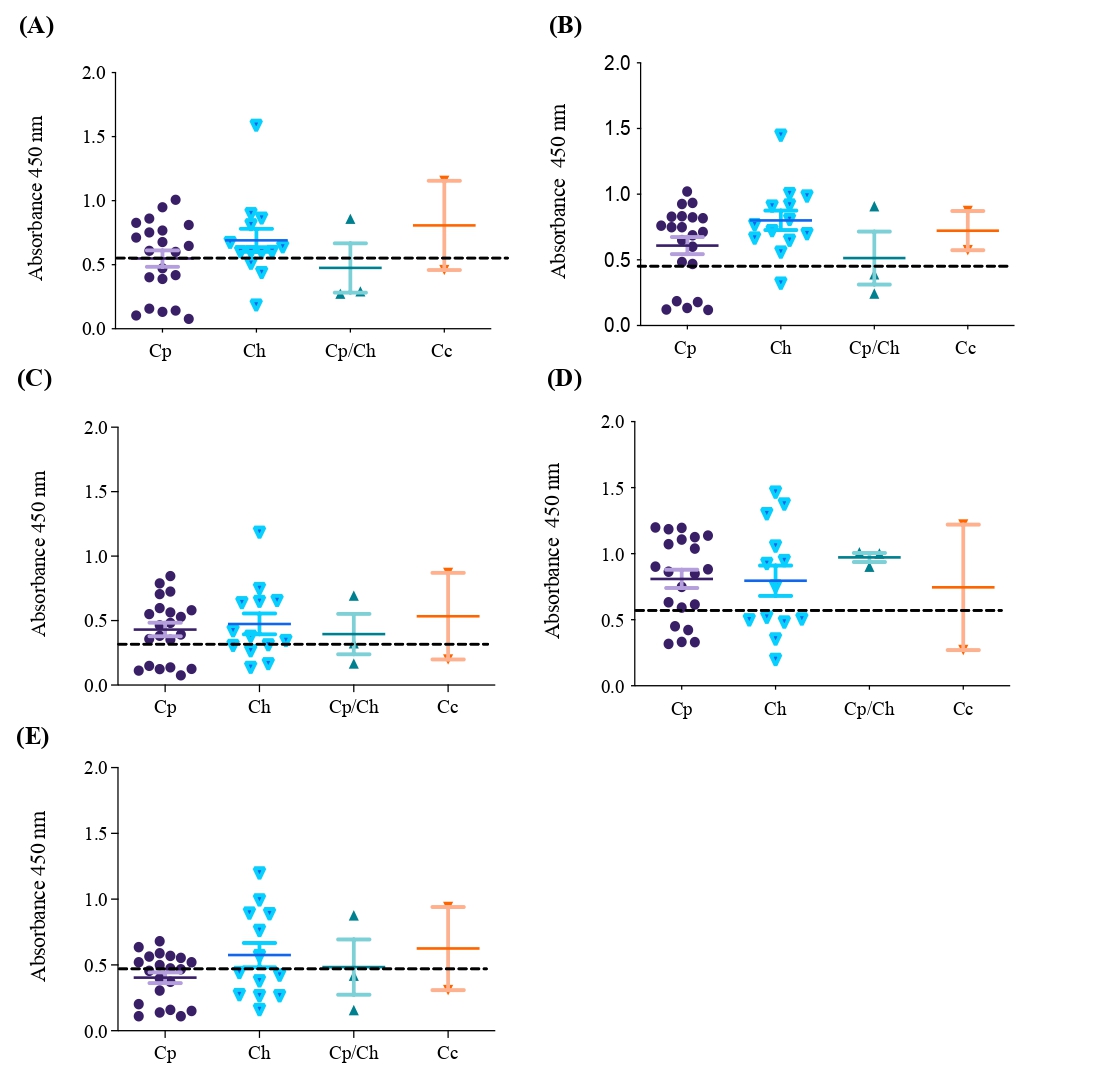

Supplement: Supplementary Figure 1 — Recognition of peptides among infecting Cryptosporidium species. The line is cut-off (average plus two standard deviations) for each peptide obtained from the average of the absorbances of the controls. Cp, C. parvum; Ch, C. hominis; Cc, C. canis. (A) Peptide A109. (B) Peptide A133. (C) Peptide V30. (D) Peptide A32. (E) Peptide R61. [file Image_1.jpeg]

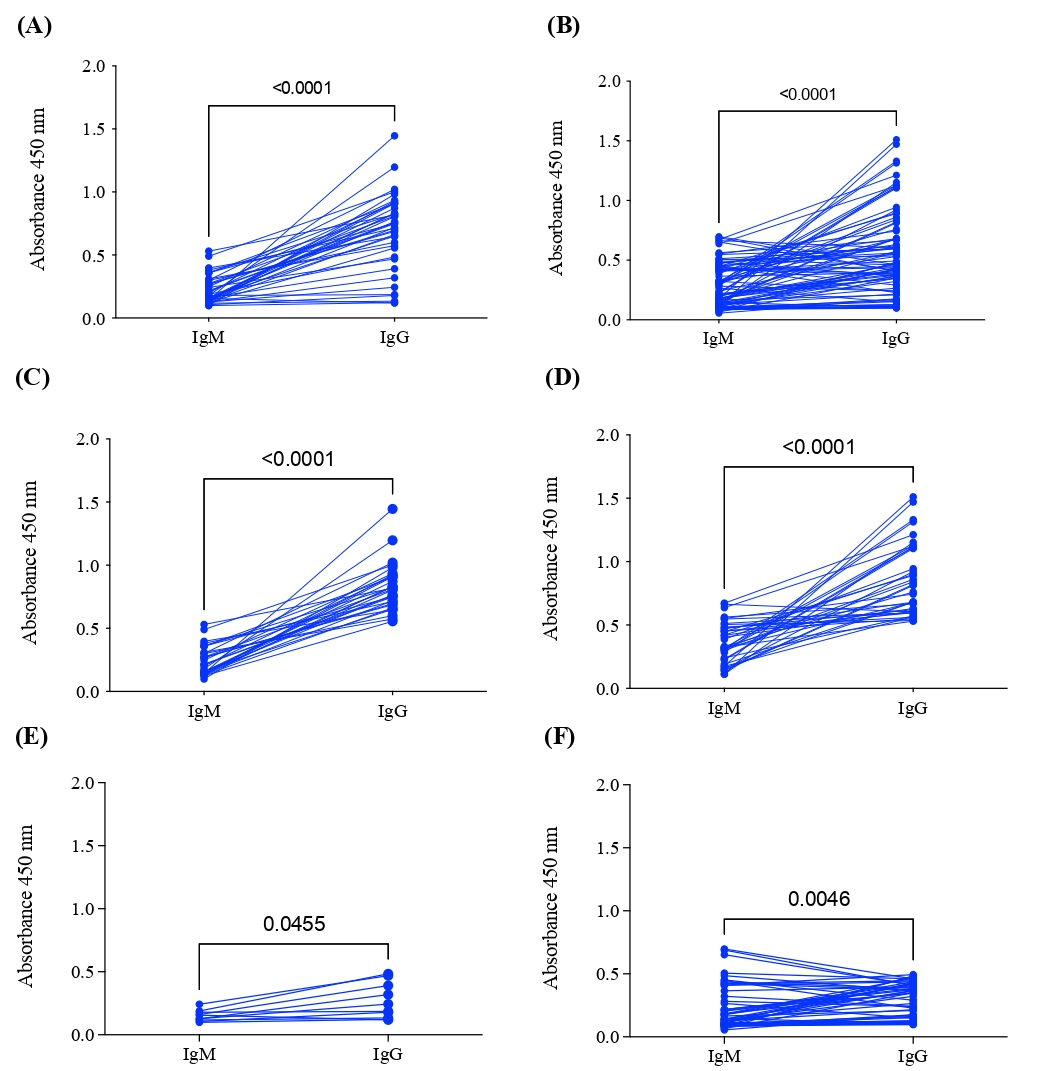

Supplement: Supplementary Figure 2 — IgM and IgG anti-Cryptosporidium A133 peptide. (A) Cases. (B) Controls. (C) Cases with IgG above the cutoff line (0.5). (D) Controls with IgG above the cut-off line (0.5). (E) Cases with IgG below the cutoff line (0.5). (F) Controls with IgG below the cutoff line. [file Image_2.jpeg]

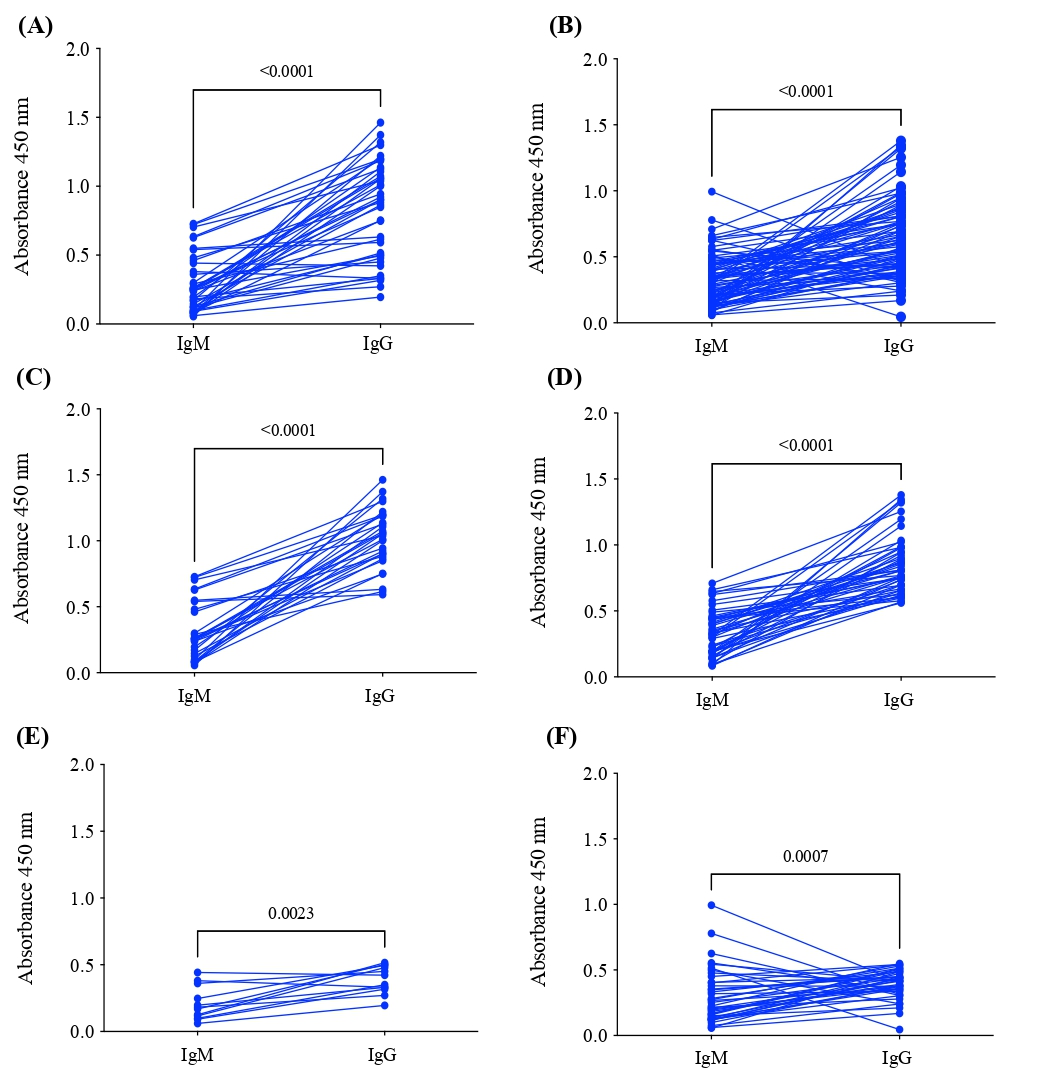

Supplement: Supplementary Figure 3 — IgM and IgG anti-Cryptosporidium A32 peptide. (A) Cases. (B) Controls. (C) Cases with IgG above the cutoff line (0.556). (D) Controls with IgG above the cut-off line (0.05). (E) Cases with IgG below the cutoff line (0.05). (F) Controls with IgG below the cut-off line. [file Image_3.jpeg]
